# Development of evidence-based guidelines for the treatment and management of periprosthetic hip infection: the INFORM guidelines

**DOI:** 10.1302/2633-1462.44.BJO-2022-0155.R1

**Published:** 2023-04-01

**Authors:** Andrew J. Moore, Vikki Wylde, Michael R. Whitehouse, Andrew D. Beswick, Nicola E. Walsh, Catherine Jameson, Ashley W. Blom

**Affiliations:** 1 University of Bristol, Bristol, UK; 2 HAS - Allied Health Professions, University of the West of England, Bristol, UK; 3 University of Sheffield Faculty of Medicine Dentistry and Health, Sheffield, UK

**Keywords:** Prosthetic infection, Guidelines, Consensus, Hip, Arthroplasty, periprosthetic hip infection, infections, joint infection, antibiotics, clinicians, debridement, antibiotics, and implant retention, primary hip arthroplasty, prosthetic joint infection, prosthetic infection, clinical study

## Abstract

**Aims:**

Periprosthetic hip-joint infection is a multifaceted and highly detrimental outcome for patients and clinicians. The incidence of prosthetic joint infection reported within two years of primary hip arthroplasty ranges from 0.8% to 2.1%. Costs of treatment are over five-times greater in people with periprosthetic hip joint infection than in those with no infection. Currently, there are no national evidence-based guidelines for treatment and management of this condition to guide clinical practice or to inform clinical study design. The aim of this study is to develop guidelines based on evidence from the six-year INFection and ORthopaedic Management (INFORM) research programme.

**Methods:**

We used a consensus process consisting of an evidence review to generate items for the guidelines and online consensus questionnaire and virtual face-to-face consensus meeting to draft the guidelines.

**Results:**

The consensus panel comprised 21 clinical experts in orthopaedics, primary care, rehabilitation, and healthcare commissioning. The final output from the consensus process was a 14-item guideline. The guidelines make recommendations regarding increased vigilance and monitoring of those at increased risk of infection; diagnosis including strategies to ensure the early recognition of prosthetic infection and referral to orthopaedic teams; treatment, including early use of DAIR and revision strategies; and postoperative management including appropriate physical and psychological support and antibiotic strategies.

**Conclusion:**

We believe the implementation of the INFORM guidelines will inform treatment protocols and clinical pathways to improve the treatment and management of periprosthetic hip infection.

Cite this article: *Bone Jt Open* 2023;4(4):226–233.

## Introduction

For many people hip arthroplasty improves pain and mobility, but some people experience complications including periprosthetic joint infection (PJI). The incidence of PJI reported within two years of primary hip arthroplasty ranges from 0.8 to 2.1%.^1-3^ The impact of PJI on patients is severe, and can result in severe pain, disability, or death.^4,5^ In the USA, it is reported that patients undergoing treatment for PJI have a two-fold increase in in-hospital mortality for each surgical admission compared to aseptic revisions, and as PJI cases often have multiple admissions, the risk of mortality is cumulative.^6^ Concerns have also been raised in regard to increasing antibiotic resistance and the growth in the number of culture-negative PJIs,^7^ which highlights the importance of increasing vigilance and prevention practices to avoid an increase in the number of PJIs.^8^ The management of PJI is complex and optimization is crucial. Most patients with PJI require either single- or two-stage revision surgery, which involves removal of the prosthesis, debridement of infected tissue, antibiotic treatment, and revision total hip arthroplasty in either one or two operations. As there are no UK standard pathways for the treatment of hip PJI, treatment is often guided by expert opinion,^9^ and other factors including availability of infrastructure (microbiology support) and the infecting organism,^10^ although use of single-stage revision is increasing.^11^

Between 2014 and 2020, theINFection ORthopaedic Management (INFORM) programme, funded by the National Institute of Health Research (RP-PG-1210 to 12005), identified ways of improving outcomes for patients with PJI.^12^ Evidence from the INFORM programme showed that risk factors for PJI are male sex, previous revision surgery, previous hip infection, diagnosis of rheumatoid arthritis, femoral bone graft during primary hip arthroplasty, smoking, history of steroid administration, obesity and significant comorbidity.^13,14^ Concerning diagnosis, qualitative research showed that patients felt their concerns about their joint often went unacknowledged and that earlier diagnosis of infection is needed.^4^ In relation to treatment, a discrete choice questionnaire showed the most valued characteristics in patient decisions about revision were the ability to engage in valued activities and a quick return to normal activity.^15^ Evidence synthesis established that early use of a more conservative debridement, antibiotics and implant retention (DAIR) strategy may be effective in over 60% of cases if the duration of infection does not exceed three weeks and if done by an appropriate surgeon,^16^ and should therefore be considered the first line of treatment in this early time period.

Qualitative research and a discrete choice experiment showed that patients have a preference for single-stage revision surgery.^15,17-19^ When two-stage surgery was undertaken, patients struggled with pain and function without articulating spacers.^20^ Patients also reported a need for more tailored physiotherapy input and psychological and social support, and experienced problems tolerating antibiotics.^4,20^ A systematic review and survey showed wide variation in provision of physical rehabilitation and a lack of psychological support.^21,22^

Evidence synthesis showed that single and two-stage revision appear equally efficacious, although single-stage had better early results and is cost-effective.^18,19^ National Joint Registry analysis showed that compared to a two-stage strategy, there is a higher rate of early re-revision after single-stage revision for hip PJI, but this equalized with time.^23^ In the INFORM randomized controlled trial, 140 patients with hip PJI were randomized to single- or two-stage revision.^24^ At 18 months after randomization, a patient-reported outcome showed no superiority of single-stage compared with two-stage revision for hip PJI. Pain, function, and stiffness were similar between randomized groups and there were no differences in reinfection or adverse events. Participants randomized to a single-stage procedure had a quicker recovery, lower costs and higher quality adjusted life years than those randomized to a two-stage procedure.^17^

At the time of writing, there are no standard UK guidelines for the treatment of hip PJI. Local pathways are often based on small local case series and observational studies.^25^ As part of a follow-on Programme Development Grant, funded by the National Institute of Health Research, the aim of this study was to mobilize evidence from the INFORM programme by using it to develop best practice guidelines which can be implemented nationally.

### Study design

We worked with expert stakeholders involved in the treatment of PJI, from across the UK, to develop best practice guidelines for hip PJI based on evidence from the INFORM programme. Evidence-based recommendations derived from the programme were evaluated by a panel of 21 expert clinical stakeholders via an online consensus questionnaire in phase 1, and subsequent consensus meeting in phase 2, similar to a Modified Nominal Group Technique.^26^ The study received Health Research Authority approval (ref: 22/HRA/0399) and University of Bristol, Faculty of Health Sciences Ethics approval (ref: 10069).

### Initial draft guideline

The initial draft guideline was developed by study team members with expertise in orthopaedic surgery, evidence synthesis, health services research, social science, physiotherapy, and knowledge mobilization. Evidence from the INFORM programme relevant to the preoperative, perioperative and postoperative patient pathway were aggregated into a table by an evidence synthesis expert, which provided the basis for the initial draft guideline. On 28 January 2022, the draft guideline was sent to the project steering committee, which comprised three consultant orthopaedic surgeons, a professor of musculoskeletal therapies, and a senior research fellow in patient experiences in trauma and musculoskeletal sciences. Further refinements were made until a draft guideline consisting of 12 statements was agreed. The draft was then reviewed by the INFORM patient and public involvement (PPI) group, which includes five people with experience of PJI.

### Sample size and recruitment

Participants were identified through participation in a previous study,^17^ and through informal and professional networks. After providing informed consent, participants completed the online questionnaire via Online Surveys (UK). Sample size was guided by the need to elicit the views of expert stakeholders in the design of guidelines, rather than statistical power.^27^

### Consensus questionnaire

The consensus questionnaire was designed to elicit opinions about the appropriateness of each guideline draft statement (Supplementary material i). Participants were asked to rate each statement from 1 to 9 ('not appropriate' to 'very appropriate'). Participants were also provided with a free-text space to explain their rating and to make suggestions for alterations or additions. All participants were assigned ID numbers, and personal data (names, contact details) were stored separately from the data. All written reports about the questionnaire data were anonymized, removing any data that may potentially be used to identify individuals. The questionnaire was piloted with a member of the research team to check functionality. The questionnaire opened on 9 March 2022 and closed on 6 April 2022.

### Stakeholder meeting

An expert stakeholder meeting was held virtually using an online video collaboration platform. The meeting was facilitated by the chief investigator (AJM) and co-chief investigator (AWB) with research team members attending. Proposed changes to the draft statements (informed by analysis of the free-text survey responses) were discussed in the meeting and then re-rated by participants. As in other co-design studies, voting was facilitated by the use of the Mentimeter interactive polling survey app, which allows stakeholder participants to use smartphones to vote anonymously.^28^ Following the meeting, those that could not attend were invited to vote by email on any amended or new statements.

### Analysis

We used the RAND/UCLA appropriateness method to determine consensus scores in the questionnaire and stakeholder meeting.^29^ A guideline statement with a median score of 1 to 3 was considered as unimportant (good to excellent consensus over lack of importance), statements with a median score of 4 to 6 as uncertain (some consensus over importance), and those with a score of 7 to 9 as important (good to excellent consensus over importance). Those guideline statements given an importance rating of 7 to 9 by ≥ 70% of participants were retained. Free-text comments were entered into a Excel spreadsheet (Microsoft, USA) and categorized according to their key content. We did not perform a full thematic or similar analysis on this qualitative data, instead, categorization in the form of tables enabled us to collate and display the information which was reviewed at a team meeting to decide whether amendments should be proposed for each statement. For statements where consensus was reached, no amendments or minor amendments were proposed, informed by the free-text comments. For statements where consensus was not reached, major amendments were proposed.

The results of the questionnaire and proposed changes were collated into a summary report and sent to participants. Any amendments were then discussed and put forward for voting at the stakeholder meeting. For those who could not attend the meeting, a summary report of the meeting was sent, and they were asked to vote on any suggested changes by email.

## Results

Overall, 21 UK-based healthcare professionals took part. There were no incomplete questionnaires. A total of 20 completed the survey, made up of orthopaedic surgeons (n = 10), rehabilitation specialists (physiotherapists and occupational therapists) (n = 5), primary care specialists (n = 4), and a healthcare commissioner (n = 1). Healthcare professionals had a declared specialist interest in the management of hip PJI with orthopaedic surgeons having experience of treating PJI ranging from five to 24 years. Primary care practitioners experience in their current roles ranged from six to 23 years, physiotherapists and occupational therapists from ten to 26 years, and a healthcare commissioner had three years’ experience. Orthopaedic consultants included members of the British Orthopaedic Association and the British Hip Society.

A total of ten of the 12 guideline statements were endorsed as appropriate by consensus (rated 7 to 9 by ≥ 70% of participants), and two did not reach consensus (rated as 7 to 9 by < 70% of participants) (please see Table I).

**Table I.** Development of the INFORM guidelines consensus survey and meeting results

Statement number on survey	Statement included in the consensus survey	Respondents who gave rating of 7 to 9 in survey, %	Modifications agreed as needed in consensus meeting	Revised/new statements developed during consensus meeting	Respondents who gave rating of 7 to 9 for revised/ new statement, %
1	Patients with postoperative complications such as slow wound healing, or unexplained pain should prompt high suspicion of infection.	80	None	Hip arthroplasty patients with postoperative complications such as slow wound healing or unexplained pain should prompt high suspicion of infection.	N/A – no revote
2	Modifiable risk factors should be optimized (e.g. diabetes control).	90	None	Modifiable risk factors should be optimised (e.g. diabetes control).	N/A – no revote
3	All patients with unexplained symptoms should be investigated for infection without delay.	60	Major modifications	All patients with persistent fluid discharge, worsening erythema or worsening pain arising from the joint should be investigated for infection.	92
4	Improve education and patient and clinician information to enable earlier recognition of signs and symptoms of infection.	70	None	Improve education and patient and clinician information to enable earlier recognition of signs and symptoms of infection.	N/A – no revote
5	Increase vigilance among primary and secondary care for patients at high risk of PJI. This includes optimizing an open door policy to allow patients to be referred back to the treating orthopaedic team promptly	95	None	Increase vigilance among primary and secondary care for patients at high risk of periprosthetic joint infection. This includes optimising an open-door policy to allow patients to be referred back to the treating orthopaedic team promptly.	N/A – no revote
6	When infection is diagnosed with well-fixed implants and DAIR is considered it should be performed promptly. This consists of a radical debridement with exchange of modular components where possible, and NOT a wound wash-out.	85	None	When infection is diagnosed with well-fixed implants, and DAIR is considered, it should be performed promptly. This consists of a radical debridement with exchange of modular components where possible, and NOT a wound wash-out.	N/A – no revote
7	Single-stage should be performed whenever surgeons believe it is feasible, and within the bounds of a well-established dialogue with the patient, characterized by a plain language explanation of treatment options, with adequate time for the patient’s questions to be answered.	85	None	Single-stage revision should be performed whenever surgeons believe it is feasible, and within the bounds of a well-established dialogue with the patient, characterized by a plain language explanation of treatment options, with adequate time for the patient’s questions to be answered.	N/A – no revote
8	Surgeons should consider the use of standard components fixed with antibiotic loaded bone cement as an articulating spacer.	70	None	Surgeons should consider the use of standard components fixed with antibiotic loaded bone cement as an articulating spacer.	N/A – no revote
9	Patients need appropriate levels of specialist physiotherapy and rehabilitation as determined through assessment from early on in their journey.	89	Minor modifications	Patients need appropriate levels of patient-centred rehabilitation as determined through assessment from early on in their journey.	86
10	Psychological and social support should be offered to all patients with infection from the point of diagnosis onwards to long-term recovery.	84	Minor modifications	Patients with infection should be asked about their need for psychological and social support and this offered from the point of diagnosis onwards to long-term recovery.	93
11	Physical aids such as wheelchairs should be provided.	84	Minor modifications	Patients should be assessed and provided with appropriate aids and equipment to support their recovery and rehabilitation.	100
12	Patients need to have antibiotics reviewed often by microbiologists until patients have a regime that is effective with tolerable side-effects.	68	Major modifications	Patients should remain under the care of an infection multidisciplinary team while on antibiotics and monitored for side-effects and tolerance.	77
13	N/A – developed as new statement during the meeting			Any patient within the first four weeks of primary joint arthroplasty, with increasing discharge or reduction in function or worsening erythema should prompt discussion with a specialist orthopaedic colleague within 48 hours.	100
14	N/A – developed as new statement during the meeting			A patient with a previously well performing hip arthroplasty, who develops symptoms consistent with infection (such as fluid discharge, new or worsening erythema and new or worsening pain) which persist for more than 48 hours, should prompt discussion with an arthroplasty specialist within 72 hours from presentation.	100

DAIR, debridement, antibiotics, and implant retention; N/A, not applicable.

The survey results and free-text comments were discussed by the research team, and minor amendments were proposed to three statements which had reached consensus and major amendments proposed to the two statements which had not reached consensus. Following the survey, 11 participants attended the stakeholder meeting, including ten who completed the survey. During the stakeholder meeting two additional statements were proposed and overall consensus was reached on 14 statements. The final INFORM guidelines are presented in Table II.

**Table II.** The INFORM guidelines for the management of hip periprosthetic joint infection.

**INCREASED VIGILANCE AND MONITORING:** *Evidence shows those at increased risk are males, people with previous revision surgery, previouship infection, hip arthroplasty for rheumatoid arthritis, or femoral bone graft during primary hip arthroplasty, smokers, people witha history of steroid administration or BMI ≥ 30 kg/m², and those with significant comorbidity (including liver disease,diabetes, chronic pulmonary disease, heart failure, and depression).* ^13,14^	
1	Hip arthroplasty patients with postoperative complications such as slow wound healing or unexplained pain should prompt high suspicion of infection.
2	Modifiable risk factors should be optimised (e.g. diabetes control).
**DIAGNOSIS: ** *Evidence shows that patients feel their concerns are often unacknowledged and an earlier diagnosis of infection is needed.* ^4^	
3	All patients with persistent fluid discharge, worsening erythema or worsening pain arising from the joint should be investigated for infection.
4	Any patient within the first four weeks of primary joint arthroplasty, with increasing discharge or reduction in function or worsening erythema should prompt discussion with a specialist orthopaedic colleague within 48 hours.
5	A patient with a previously well performing hip arthroplasty, who develops symptoms consistent with infection (such as fluid discharge, new or worsening erythema and new or worsening pain) which persist for more than 48 hours, should prompt discussion with an arthroplasty specialist within 72 hours from presentation.
6	Improve education and patient and clinician information to enable earlier recognition of signs and symptoms of infection.
7	Increase vigilance among primary and secondary care for patients at high risk of periprosthetic joint infection. This includes optimising an open-door policy to allow patients to be referred back to the treating orthopaedic team promptly.
**TREATMENT:** **Debridement, antibiotics, and implant retention (DAIR)** *Evidence shows that DAIR works well if done early with thorough debridement by an appropriate surgeon.* ^16^	
8	When infection is diagnosed with well-fixed implants, and DAIR is considered, it should be performed promptly. This consists of a radical debridement with exchange of modular components where possible, and NOT a wound wash-out.
**REVISION:** *Evidence shows that patients have a preference for single-stage surgery which is equally efficacious to two-stage surgery and patients have earlier recovery* ^15,17-19,24^	
9	Single stage revision should be performed whenever surgeons believe it is feasible, and within the bounds of a well-established dialogue with the patient, characterised by a plain language explanation of treatment options, with adequate time for the patient’s questions to be answered.
10	Surgeons should consider the use of standard components fixed with antibiotic loaded bone cement as an articulating spacer.
**POSTOPERATIVE MANAGEMENT:** *Evidence shows that when surgery is undertaken, patients struggle with function and report a need for tailored physiotherapy input.* ^4,20^	
11	Patients need appropriate levels of patient-centred rehabilitation as determined through assessment from early on in their journey.
12	Patients with infection should be asked about their need for psychological and social support and this offered from the point of diagnosis onwards to long-term recovery.
13	Patients should be assessed and provided with appropriate aids and equipment to support their recovery and rehabilitation.
14	Patients should remain under the care of an infection multidisciplinary team while on antibiotics and monitored for side-effects and tolerance.

The guidelines are structured to correspond with the stages of disease and management, and each guideline statement is based on evidence from the INFORM programme. After reviewing the final guideline, the project steering committee, members of the Executive and Committees of the British Hip Society, and the British Infection Society provided statements of support. The online version of the guideline is available in Supplementary material iii.

A full description of the voting process can be found in Supplementary material ii.

## Discussion

This is the first study to develop consensus and evidence-based guidelines on the treatment of hip PJI which considers the view of orthopaedic surgeons, rehabilitation specialists, primary care experts, and health commissioners. The guideline statements are based on high-quality evidence from the INFORM programme. PJI presents as a multifaceted and highly detrimental outcome, and the guidelines address each stage from prevention to postoperative management, and are relevant to primary, secondary, and tertiary care. The strength of these guidelines is the robust evidence base used to inform them, and the collaborative and inclusive approach to agreeing the prioritization of the evidence to inform best practice.

The primary sections of the guideline address the need for increased vigilance and monitoring of those at increased risk of infection, and the need to reduce modifiable risk factors where possible. The guidelines on diagnosis of PJI address the warning signs of early and late infections and the need for prompt action, including recommended timelines in which this should happen. Improving education and patient and clinician information should enable earlier recognition of infection, and increased vigilance among primary and secondary care for patients at higher risk of infection, including an open-door policy to expediate referral back to the treating orthopaedic team. Treatment includes recommended early use of DAIR, and, where possible, single-stage revision, with standard components fixed with antibiotic-loaded cement as an articulating spacer. Postoperative management addresses the need for patient-centred physical rehabilitation from early in the patient’s journey, with psychological and social support offered from point of diagnosis onwards to longer-term recovery. It is also recommended that patients on antibiotics remain in the care of an infection multidisciplinary team.

While surgical strategies have evolved and recent shifts in practice are already aligned with many of these guidelines,^30,31^ psychological support is increasingly seen to be crucial for PJI patients and may stand out as the most challenging to address. Evidence from the INFORM programme described the psychological impact on patients and their unmet needs,^4,21,22,32^ but more recent studies have also identified this gap in provision and the need for more research in this area.^33-39^ The provision of appropriate levels of patient-centred physical and psychological rehabilitation is likely to require further resourcing to optimize these outcomes, and evidence-based guidelines strengthen the rationale for these resources being made available.

A limitation to the study is that the guideline was formulated on the evidence base from the INFORM programme, which, while comprehensive, had limitations. For example, although extensive efforts were made to ensure wide sampling and generalizability in the research studies in INFORM, we may not have reached underserved communities. Also, the research focuses on those that have experienced a complication predicated on receiving the primary intervention, which may exclude some patients.

These guidelines can be used as the basis to update local treatment pathways and protocols. While evidence-based guidelines do not necessarily translate into consistent change, recent studies have shown that the strength of evidence underpinning guidelines, dissemination with surgeon education and collaboration, and reinforcing feedback are most effective in achieving sustainable change.^40^ To ensure that the guidelines can be effectively implemented, we have developed a learning collaborative, consisting of orthopaedic surgeons and healthcare professionals from orthopaedic centres across the UK, to implement the INFORM guidelines. Learning collaboratives have previously been used successfully in diabetes,^41^ HIV,^42^ childhood asthma,^43^ and rheumatoid arthritis.^44^ They provide a way to achieve sustainable change within a healthcare area, by engaging collaborators and agreeing on best evidence-based practices and then sharing learning and experiences of implementing changes across centres. The implementation of these guidelines has the potential to improve treatment pathways and care for periprosthetic hip joint infection nationally.

**Take home message**

- The implementation of these best practice guidelines has the potential to improve treatment pathways and care for prosthetic hip joint infection nationally.

- The strength of these guidelines is the robust evidence base used to inform them, and the collaborative and inclusive approach to agreeing the prioritization of the evidence to inform best practice.

## References

[1] Choi HJ Adiyani L Sung J et al. Five-year decreased incidence of surgical site infections following gastrectomy and prosthetic joint replacement surgery through active surveillance by the Korean Nosocomial Infection Surveillance System J Hosp Infect 2016 93 4 339 346 26944901 10.1016/j.jhin.2015.12.021

[2] Lindgren V Gordon M Wretenberg P Kärrholm J Garellick G Deep infection after total hip replacement: a method for national incidence surveillance Infect Control Hosp Epidemiol 2014 35 12 1491 1496 25419771 10.1086/678600

[3] Huotari K Peltola M Jämsen E The incidence of late prosthetic joint infections: a registry-based study of 112,708 primary hip and knee replacements Acta Orthop 2015 86 3 321 325 25813645 10.3109/17453674.2015.1035173 PMC4443453

[4] Moore AJ Blom AW Whitehouse MR Gooberman-Hill R Deep prosthetic joint infection: a qualitative study of the impact on patients and their experiences of revision surgery BMJ Open 2015 5 12 e009495 10.1136/bmjopen-2015-009495 PMC4679895 26644124

[5] Hunter G Dandy D The natural history of the patient with an infected total hip replacement J Bone Joint Surg Br 1977 59-B 3 293 297 10.1302/0301-620X.59B3.893507 893507

[6] Shahi A Tan TL Chen AF Maltenfort MG Parvizi J In-hospital mortality in patients with periprosthetic joint infection J Arthroplasty 2017 32 3 948 952 27816369 10.1016/j.arth.2016.09.027

[7] Gallo J Kolar M Dendis M et al. Culture and PCR analysis of joint fluid in the diagnosis of prosthetic joint infection New Microbiol 2008 31 1 97 104 18437847

[8] Iannotti F Prati P Fidanza A et al. Prevention of periprosthetic joint infection (PJI): A clinical practice protocol in high-risk patients Trop Med Infect Dis 2020 5 4 186 33322463 10.3390/tropicalmed5040186 PMC7768381

[9] Zimmerli W Trampuz A Ochsner PE Prosthetic joint infections N Engl J Med 2004 351 16 1645 1654 15483283 10.1056/NEJMra040181

[10] Moore AJ Blom AW Whitehouse MR Gooberman-Hill R Managing uncertainty - a qualitative study of surgeons’ decision-making for one-stage and two-stage revision surgery for prosthetic hip joint infection BMC Musculoskelet Disord 2017 18 1 154 28403859 10.1186/s12891-017-1499-z PMC5388991

[11] Lenguerrand E Whitehouse MR Beswick AD Jones SA Porter ML Blom AW Revision for prosthetic joint infection following hip arthroplasty: Evidence from the National Joint Registry Bone Joint Res 2017 6 6 391 398 28642256 10.1302/2046-3758.66.BJR-2017-0003.R1 PMC5492333

[12] Blom AW Beswick AD Burston A et al. Infection after total joint replacement of the hip and knee: research programme including the INFORM RCT Programme Grants Appl Res 2022 10 10 1 190 36469653

[13] Kunutsor SK Whitehouse MR Blom AW Beswick AD INFORM team Patient-related risk factors for periprosthetic joint infection after total joint arthroplasty: A systematic review and meta-analysis PLoS One 2016 11 3 e0150866 26938768 10.1371/journal.pone.0150866 PMC4777569

[14] Lenguerrand E Whitehouse MR Beswick AD et al. Risk factors associated with revision for prosthetic joint infection after hip replacement: a prospective observational cohort study Lancet Infect Dis 2018 18 9 1004 1014 30056097 10.1016/S1473-3099(18)30345-1 PMC6105575

[15] Carroll FE Gooberman-Hill R Strange S Blom AW Moore AJ What are patients’ preferences for revision surgery after periprosthetic joint infection? A discrete choice experiment BMJ Open 2020 10 1 e031645 10.1136/bmjopen-2019-031645 PMC7044986 31969360

[16] Kunutsor SK Beswick AD Whitehouse MR Wylde V Blom AW Debridement, antibiotics and implant retention for periprosthetic joint infections: A systematic review and meta-analysis of treatment outcomes J Infect 2018 77 6 479 488 30205122 10.1016/j.jinf.2018.08.017

[17] Blom AW Lenguerrand E Strange S et al. Clinical and cost effectiveness of single stage compared with two stage revision for hip prosthetic joint infection (INFORM): pragmatic, parallel group, open label, randomised controlled trial BMJ 2022 379 e071281 36316046 10.1136/bmj-2022-071281 PMC9645409

[18] Kunutsor SK Whitehouse MR Blom AW Beswick AD INFORM team Re-infection outcomes following one- and two-stage surgical revision of infected hip prosthesis: A systematic review and meta-analysis PLoS One 2015 10 9 e0139166 26407003 10.1371/journal.pone.0139166 PMC4583275

[19] Kunutsor SK Whitehouse MR Blom AW et al. One- and two-stage surgical revision of peri-prosthetic joint infection of the hip: a pooled individual participant data analysis of 44 cohort studies Eur J Epidemiol 2018 33 10 933 946 29623671 10.1007/s10654-018-0377-9 PMC6153557

[20] Palmer CK Gooberman-Hill R Blom AW Whitehouse MR Moore AJ Post-surgery and recovery experiences following one- and two-stage revision for prosthetic joint infection-A qualitative study of patients’ experiences PLoS One 2020 15 8 e0237047 32745086 10.1371/journal.pone.0237047 PMC7398523

[21] Kunutsor SK Beswick AD Peters TJ et al. Health care needs and support for patients undergoing treatment for prosthetic joint infection following hip or knee arthroplasty: A systematic review PLoS One 2017 12 1 e0169068 28046049 10.1371/journal.pone.0169068 PMC5207523

[22] Moore AJ Whitehouse MR Gooberman-Hill R et al. A UK national survey of care pathways and support offered to patients receiving revision surgery for prosthetic joint infection in the highest volume NHS orthopaedic centres Musculoskeletal Care 2017 15 4 379 385 28332761 10.1002/msc.1186 PMC5763340

[23] Lenguerrand E Whitehouse MR Kunutsor SK et al. Mortality and re-revision following single-stage and two-stage revision surgery for the management of infected primary knee arthroplasty in England and Wales : evidence from the National Joint Registry Bone Joint Res 2022 11 10 690 699 36177603 10.1302/2046-3758.1110.BJR-2021-0555.R1 PMC9582862

[24] Strange S Whitehouse MR Beswick AD et al. One-stage or two-stage revision surgery for prosthetic hip joint infection--the INFORM trial: a study protocol for a randomised controlled trial Trials 2016 17 90 26883420 10.1186/s13063-016-1213-8 PMC4756538

[25] Khan U Perks G Morgan-Jones R et al. Pathways in Prosthetic Joint Infection Oxford University Press 2018

[26] Levine DA Saag KG Casebeer LL Colon-Emeric C Lyles KW Shewchuk RM Using a modified nominal group technique to elicit director of nursing input for an osteoporosis intervention J Am Med Dir Assoc 2006 7 7 420 425 16979085 10.1016/j.jamda.2006.05.004 PMC1839832

[27] Cantrill JA Sibbald B Buetow S The Delphi and nominal group techniques in health services research Int J Pharm Pract 2011 4 2 67 74

[28] Schimmer R Orre C Öberg U Danielsson K Hörnsten Å Digital person-centered self-management support for people with type 2 diabetes: Qualitative study exploring design challenges JMIR Diabetes 2019 4 3 e10702 31538941 10.2196/10702 PMC6754678

[29] Fitch K Bernstein SJ Aguilar MD Burnand B The RAND/UCLA Appropriateness Method User’s Manual Santa Monica, California, USA RAND Corporation 2001

[30] Liechti EF Neufeld ME Soto F et al. Favourable outcomes of repeat one-stage exchange for periprosthetic joint infection of the hip Bone Joint J 2022 104-B 1 27 33 34969284 10.1302/0301-620X.104B1.BJJ-2021-0970.R1

[31] Biddle M Kennedy JW Wright PM Ritchie ND Meek RMD Rooney BP Improving outcomes in acute and chronic periprosthetic hip and knee joint infection with a multidisciplinary approach Bone Jt Open 2021 2 7 509 514 34247508 10.1302/2633-1462.27.BJO-2021-0064.R1 PMC8325970

[32] Mallon CM Gooberman-Hill R Moore AJ Infection after knee replacement: a qualitative study of impact of periprosthetic knee infection BMC Musculoskelet Disord 2018 19 1 352 30285692 10.1186/s12891-018-2264-7 PMC6167863

[33] Furdock RJ Jilakara B Moon TJ et al. Depression Is transiently increased in patients undergoing two-stage revision arthroplasty Arthroplast Today 2022 13 136 141 35106350 10.1016/j.artd.2021.10.014 PMC8784305

[34] Hegde V Bracey DN Johnson RM Dennis DA Jennings JM Increased prevalence of depressive symptoms in patients undergoing revision for periprosthetic joint infection Arthroplast Today 2022 13 69 75 34977309 10.1016/j.artd.2021.09.011 PMC8685908

[35] Knebel C Menzemer J Pohlig F et al. Periprosthetic joint infection of the knee causes high levels of psychosocial distress: A prospective cohort study Surg Infect (Larchmt) 2020 21 10 877 883 32282286 10.1089/sur.2019.368

[36] Lueck E Schlaepfer TE Schildberg FA et al. The psychological burden of a two-stage exchange of infected total hip and knee arthroplasties J Health Psychol 2022 27 2 470 480 32840382 10.1177/1359105320948583

[37] Schwartz AM Wilson JM Farley KX Bradbury TL Guild GN New-onset depression after total knee arthroplasty: Consideration of the at-risk patient J Arthroplasty 2021 36 9 3131 3136 33934951 10.1016/j.arth.2021.04.008

[38] Walter N Rupp M Baertl S Hinterberger T Alt V Prevalence of psychological comorbidities in bone infection J Psychosom Res 2022 157 110806 35367917 10.1016/j.jpsychores.2022.110806

[39] Walter N Rupp M Hierl K et al. Long-term patient-related auality of life after knee periprosthetic joint infection J Clin Med 2021 10 5 907 33668957 10.3390/jcm10050907 PMC7956307

[40] Arroyo NA Gessert T Hitchcock M et al. What promotes surgeon practice change? A scoping review of innovation adoption in surgical practice Ann Surg 2021 273 3 474 482 33055590 10.1097/SLA.0000000000004355 PMC10777662

[41] Benedetti R Flock B Pedersen S Ahern M Improved clinical outcomes for fee-for-service physician practices participating in a diabetes care collaborative Jt Comm J Qual Saf 2004 30 4 187 194 15085784 10.1016/s1549-3741(04)30020-1

[42] Landon BE Wilson IB McInnes K et al. Effects of a quality improvement collaborative on the outcome of care of patients with HIV infection: the EQHIV study Ann Intern Med 2004 140 11 887 896 15172903 10.7326/0003-4819-140-11-200406010-00010

[43] Homer CJ Forbes P Horvitz L Peterson LE Wypij D Heinrich P Impact of a quality improvement program on care and outcomes for children with asthma Arch Pediatr Adolesc Med 2005 159 5 464 469 15867121 10.1001/archpedi.159.5.464

[44] Solomon DH Losina E Lu B et al. Implementation of treat-to-target in rheumatoid arthritis through a learning collaborative: Results of a randomized controlled trial Arthritis Rheumatol 2017 69 7 1374 1380 28512998 10.1002/art.40111 PMC5489379

